# Young people who have fallen through the mental health transition gap: a qualitative study on primary care support

**DOI:** 10.3399/BJGP.2021.0678

**Published:** 2022-05-04

**Authors:** Rebecca Appleton, Joelle Loew, Faraz Mughal

**Affiliations:** National Institute for Health Research (NIHR) Mental Health Policy Research Unit, Division of Psychiatry, UCL, London.; Department of Languages and Literatures, University of Basel, Switzerland; lecturer in English business communication, Lucerne University of Applied Sciences and Arts, Switzerland.; School of Medicine, Keele University, Keele; honorary clinical research fellow, Unit of Academic Primary Care, University of Warwick, Coventry; affiliate, NIHR Greater Manchester Patient Safety Translational Research Centre, Keele University, Keele.

**Keywords:** transition, young people, mental health, qualitative research: general practice, primary care

## Abstract

**Background:**

Owing to poor continuity of care between child and adult mental health services, young people are often discharged to their GP when they reach the upper boundary of child and adolescent mental health services (CAMHS). This handover is poorly managed, and GPs can struggle to support young people without input from specialist services. Little is known about young people’s experiences of accessing mental health support from their GP after leaving CAMHS.

**Aim:**

To explore the experiences and perspectives of young people and the parents/carers of young people receiving primary care support after CAMHS and to identify barriers and facilitators to accessing primary care.

**Design and setting:**

Qualitative study with young people and parents in two English counties: London and West Midlands.

**Method:**

Narrative interviews were conducted with 14 young people and 13 parents who had experienced poor continuity of care after reaching CAMHS transition boundary. Data were analysed using reflexive thematic analysis.

**Results:**

Three themes were identified: unmet mental health needs, disjointed care, and taking responsibility for the young person’s mental health care. Barriers included the perception that GPs couldn’t prescribe certain medication, anxiety caused by the general practice environment, and having to move to a new practice at university. Young people’s positive experiences were more likely to include having a long-term relationship with their GP and finding that their GP made time to understand their needs and experiences.

**Conclusion:**

GPs could help to meet the unmet needs of young people unable to access specialist mental health services after leaving CAMHS. There is a need for comprehensive handover of care from CAMHS to GPs, which could include a joint meeting with the young person and a member of the CAMHS team. Future research should focus on interventions which improve continuity of care for young people after leaving CAMHS, and collaborative working across community mental health services.

## INTRODUCTION

It has been widely reported that young people receiving care at child and adolescent mental health services (CAMHS) struggle to receive continuity of mental health care when they reach the upper age limit of CAMHS when they are aged 16–18 years.^1^^–^^6^ If there is an ongoing mental health clinical need, care should be transferred to an adult mental health service (AMHS) through a managed process known as ‘transition’.^7^ However, it is known that only around one-quarter of young people transition to AMHS;^3^ therefore, the majority of young people need to access support elsewhere. If a young person is not referred to AMHS once they reach the CAMHS transition boundary despite still being unwell, they are said to have ‘fallen through the gap’ between services. Young people who fall through the gap have reported anxiety and frustration caused by a lack of continuity of care, especially at a time where they are likely to experience several concurrent life transitions.^8^ For some young people, a lack of mental health care can result in increased distress and feeling unable to cope on their own.^9^

In many cases, young people are discharged to their GP when they reach the upper age limit of CAMHS.^2^^.^^3^ This process is far from being standardised,^2^^,^^10^ with GPs reporting the absence of a handover and little communication from mental health services.^11^ This is in contradiction to National Institute for Health and Care Excellence (NICE) guidance, which states that GPs should be involved in transition planning, especially if the young person does not meet the criteria for AMHS.^12^ A combination of factors including the scarcity of available services, high eligibility thresholds, and long waiting lists contribute to difficulties in referring young people to AMHS,^11^ which can lead to GPs becoming responsible for the young person’s care. GPs have also reported struggling to refer young people to AMHS.^11^

There is little research on the experiences of young people who access mental health support from their GP after leaving CAMHS. Those who fall through the gap are currently underrepresented in the literature, as they can be difficult to recruit to studies owing to not being under a mental health service. This qualitative study had the opportunity to interview young people who have fallen through the transition gap because it formed part of a longitudinal study^13^ which followed up young people for 2 years after leaving CAMHS.

Experiences of falling through the gap with a focus on accessing adult mental health support have been published recently.^14^ During the analysis for this previous study, it became clear that GPs played an important role in the care of young people after leaving CAMHS, and that this warranted further investigation. This study therefore aimed to answer the following questions:

- What are the experiences of young people who have fallen through the gap and their parents in receiving primary care support for their mental health?
- What are the barriers and facilitators to accessing mental health care in primary care for young people who have fallen through the gap?

**Table:** How this fits in

Young people who reach the upper age limit of child and adolescent mental health services (CAMHS) are often discharged to their GP while still requiring support for their mental health; however, little is currently known about their experiences of accessing mental health care from their GP at this point. This study explored the perspectives of young people and parents after leaving CAMHS. Young people described mixed experiences of accessing mental health support from their GP, with facilitators including GPs taking the time to listen and understand their needs, and a long-term relationship with the same GP.

## METHOD

This study is linked to a wider project which explored transition across eight different European countries and conducted assessments with young people at four timepoints over a 2-year period.^13^ This study included UK participants only and informed written consent was obtained from all participants. This research is reported according to the Standards for Reporting Qualitative Research.^15^

### Eligibility

Young people were eligible for inclusion if they had fallen through the gap between services and had a diagnosis of a neurodevelopmental, anxiety, or personality disorder, or depression. These diagnoses were chosen as they are the diagnostic groups most likely to fall through the gap,^1^ or in the case of emerging personality disorder, owing to previous contradictory findings regarding likelihood of transition.^1^^,^^16^ Young people were said to have fallen through the gap between services if they were not referred to an AMHS or a community mental health service after CAMHS despite having a clinical need, or if they were referred to another service only to be later discharged with an ongoing clinical need. Clinical need was measured by a score of ≥2 on the Health of the Nation Outcomes Scale for Children and Adolescents (HoNOSCA: a measure of health and social functioning) questions^17^ relating to psychological impairment. If a Young people met the inclusion criteria then their parent or carer was also invited to take part. There were no age-related eligibility criteria for this study as the CAMHS transition boundary varied across NHS trusts; therefore, young people would have been between 17 and 21 at the time of interview.

### Recruitment

A sample size of 12–15 young people and 12–15 parents was aimed for based on the principle of maximum variation sampling.^18^ Young people and parents were recruited using purposeful stratified sampling^19^ to generate a diverse sample across locations, diagnosis, sex, and ethnicity. Participants were first introduced to the study via a posted study information pack and invitation letter with details of how to respond if they were interested in taking part. If there had been no response, this was followed up by a phone call or text message 2 weeks later to ask if they wanted to be involved in the study.

### Data collection

All interviews were conducted by a female researcher with previous experience and training in qualitative research using a narrative approach,^20^ followed by purposeful questioning.^21^ A narrative approach was chosen as it encourages people to tell their stories, and is therefore a useful method for giving a voice to a population who have not been heard from before.^22^ Interviews took place over the phone or in person, depending on participant preference, and were audiorecorded and transcribed verbatim. A sample interview topic guide is shown below in Box 1. A reflexive research diary was kept throughout data collection and analysis to reflect on and minimise the impact of researcher bias.

**Box 1.** An example narrative interview structure with purposeful questioning

**Example interview structure** Can you tell me about the time when you/your son or daughter first started receiving care at [name of service]? What was your/their experience at CAMHS like? How did you/they experience the end of care at [name of service]? What has happened since you/they left [name of service] in terms of accessing other services? [Questions on topics discussed in interview if more information is needed] Is there anything else you would like to add which we haven’t talked about yet today?

*CAMHS = child and adolescent mental health services.*

### Analysis

Data in the form of interview transcripts were analysed using the reflexive thematic analysis method of Braun and Clark.^23^ Data were coded separately, and then all codes and emerging categories were discussed by all authors and refined over several cycles, before all authors agreed on the final themes. The research team has extensive experience in qualitative research in different disciplines (psychology, linguistics, and general practice) which increases the trustworthiness of the findings.^24^

## RESULTS

Of the 15 young people and 15 parents interviewed, 14 young people and 13 parents mentioned contact with their GP at the transition boundary; therefore, these participant transcripts were included for analysis. This resulted in 16 individual transition stories (in 11 cases, the young person and the parent were interviewed either together or separately, three young people took part in the study without their parent, and two parents took part in the study without their child).

Young people and parents who took part belonged to eight different NHS Mental Health Trusts across the West Midlands and London. All interviews were conducted between February and April 2019. Full demographic details for the young people linked with each transition story are presented in Table 1. Demographic details for parents were not recorded. Individual interviews ranged from 14–81 minutes (average = 36 minutes), while joint interviews ranged from 40–82 minutes (average = 56 minutes).

**Table 1.** Demographic details of the young people linked with each transition story (*n* = 16)

**Age in years, mean**	19.31

**Sex *n*, (%)**	
Female	9 (56.3)
Male	7 (43.8)

**Ethnicity *n*, (%)**	
White British	15 (93.8)
British Asian	1 (6.2)

**Diagnosis *n*, (%)**	
Mood and anxiety disorders	7 (43.8)
Comorbid autism and mood/anxiety disorder	4 (25.0)
Autism	2 (12.5)
ADHD	1 (6.3)
Other^a^	2 (12.5)

**Time since transition**	
1–2 years	13 (81.2)
3–4 years	3 (18.8)

**Current employment status**	
University student	9 (56.3)
College/sixth-form student	2 (12.5)
Full time employment	2 (12.5)
Not in education, employment, or training	3 (18.8)

**Current living situation**	
Family home	7 (43.8)
University accommodation and family home	7 (43.8)
Moved out of family home	2 (12.5)

a *Autism or OCD with comorbidities including substance abuse and speech/communication disorder. ADHD = attention deficit hyperactivity disorder. OCD = obsessive compulsive disorder.*

Three main themes were generated; they are described in turn below with illustrative participant quotations.

### Unmet mental health needs

Young people often stated difficulties in accessing care which met their mental health needs after reaching the upper age limit of CAMHS. In some cases, this was because young people were unable to access the specialist mental health support they needed. There were instances where young people went back to their GP to ask for a referral elsewhere, only for this referral to be rejected by the specialist service:

*‘… and then my GP referred me, and that was basically just to tick the boxes and send off the request form, and she sent it off and basically ticked everything that meant that they’d definitely see me, and they just rejected my case, they didn’t even look at me.’*

(Young person [YP]1, F [female], aged 20 years, depression and anxiety)

While this young person (YP1, quoted above) made the assumption that the specialist service ‘ *just rejected my case’*, which cannot be confirmed, they report that even though their GP tried to ‘ *tick everything’* so as to ensure the patient would be seen, the referral was rejected. Where young people were unable to access specialist support, their GP became the main provider of mental health care. However, this raises the issue of GP capability, as some young people felt that some GPs did not have the appropriate understanding of mental illness to provide them with the necessary support. This is an identified barrier to young people seeking mental health support from their GP at the transition boundary:

*‘And then you get there and they’re just like — I remember one doctor was quite harsher one day, he just said “Oh you’ll feel down sometimes,” and that was it.’*

(YP13, F, 20 years, comorbid autism and mood/anxiety disorder)

Further to this, some young people also struggled with the environment of a GP practice, which caused them anxiety and was another identified barrier to accessing mental health support from their GP:

*‘And it was like, the waiting when you’re not feeling mentally well, that was hard. Because you’re just sat there panicking, and you’re surrounded by people who are coughing and genuinely sick.’*

(YP13, F, 20 years, comorbid autism and mood/anxiety disorder)

Moreover, some young people and parents did not perceive their GP as having the correct expertise to support their mental health needs, which put them off visiting their GP for mental health support:

*‘I mean there isn’t anything, it needed to be done that way so I knew where I could go if I needed support for* [name] *and there wasn’t anything, apart from my GP. And then my GP would have to try and source it, and she doesn’t really know either.’*

(Parent/Carer [P/C]1, anxiety)

*‘And I don’t really like speaking to a GP about it, which obviously I was left to do, because, I don’t know, I don’t mind speaking to them about medicine alone, but I’m not very good at opening up, because I’m like “Well that’s not what you’re really here for, is it?”’*

(YP6, F, 18 years, comorbid neurodevelopmental/anxiety)

This also included participants describing instances where they were unsure of the GP’s ability to prescribe their medication:

*‘I don’t know how much control they* [GPs] *have with the Concerta* [methylphenidate] *tablet itself … I don’t know much about what GPs can and can’t do.’*

(YP8, [M] male, 19 years, attention deficit hyperactivity disorder [ADHD])

### Disjointed care

**Figure 1.**
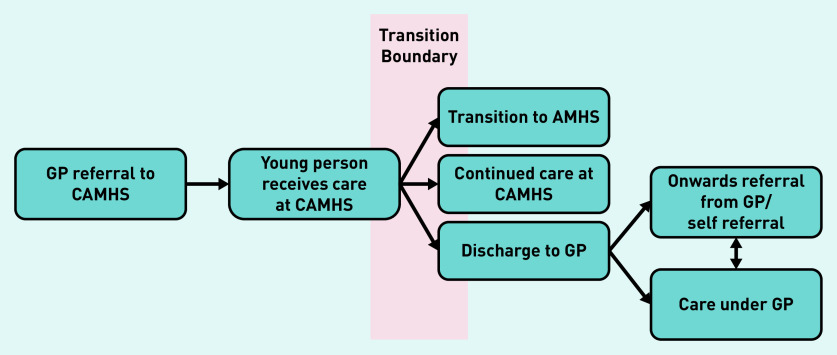
*A flow chart of the care pathway from child to adult mental health services. AMHS = adult mental health services. CAMHS = child and adolescent mental health services.*

Young people and parents perceived mental health services as being disjointed, which makes it difficult for service users, their parents, and in some cases their clinicians to understand care pathways or know where they can access mental health support. This disjointed nature of care is also reflected in the way young people talked about services, as they often contradicted themselves with regard to identifying relevant stages in the care pathway, or members of their care team. A diagram of the care pathway is shown in Figure 1.

In some cases, disjointed services led to poor continuity of care for young people. Where the young people did not meet the threshold for continued care at AMHS, CAMHS often discharged young people to their GP for the GP to make a referral elsewhere. This process was not always managed well:

*‘And* [CAMHS said] *we’d have to go back to our doctor* [GP]. *We went back to the doctor, the doctor didn’t really have a clue what we were talking about, because we said “Well, what happens now?” And they didn’t really give us much information at all, so we just felt like we were just, left.’*

(P/C3, depression)

This creates another step in the care pathway and in some cases can result in lengthy delays for young people to access further mental health support after leaving CAMHS. This is also often the first time that the GP has been involved in the young person’s care since they first presented to CAMHS, as it is often the GP who makes the initial referral, so they may not be up to date about that young person’s needs:

‘*My son’s going to come, he’s got this problem, but just bear in mind that it may be difficult for him to answer certain questions, obviously, because he’s got ASD* [autism spectrum disorder] *, and they* [GP] *went “What?”, the GP said to me “What?”, and I said “He’s got ASD”. It wasn’t on his records.’*

(P/C12, comorbid autism and obsessive-compulsive disorder [OCD])

Young people also experienced disjointed care owing to changing to a new GP if they moved to university. This led to some anxiety as the new GP may not know about their mental health history, and was perceived by young people and parents as a barrier to accessing mental health support:

*‘Because I don’t know if the doctor at university knows anything about her history, and also I suppose, given the history, if the records are passed on, should somebody be alerted, that knows, you know, that she’s somebody that — do you know what I mean, I just feel like there just doesn’t seem to be any sort of cohesion.’*

(P/C14, speech/communication disorder and OCD)

In addition to this, a lack of joined-up care in the transition process is also reflected in the use of the terminology to describe the process of moving through the care pathway after CAMHS. That is, when young people do not meet the threshold for care at AMHS, they are ‘discharged to their GP’, regardless of whether they still require mental health support. This ‘discharge’ implies that a process has come to an end, which stands in contrast to the ongoing process known as transition that aims to involve young people in decisions around their care. In fact, young people and their parents often remove their own agency when describing these processes; for example, *‘they just passed her to the GP* ’ (P/C1, anxiety), or *‘they just said “we’re going to refer you back to your GP”, and that was it’* (P/C3, depression), indicating that rather than being a managed, active process, the transfer of the care to the GP is conducted in a way which results in poor continuity of care for the young person.

### Taking responsibility for the young person’s mental health care

This theme relates to the ongoing negotiation of who is responsible for ensuring that young people receive mental health care if needed, once they reach the upper age limit of CAMHS.

In several cases, young people in this study were supported by their parents to access GP support. This is a facilitator in accessing GP care at this point, but may become a barrier when the young person is aged over 18 years:

*‘I was saying “Come on, you need to ring your GP”, whereas before I could have done that for her. Now, because she’s 18, she’s got to do it herself, and it’s much more difficult.’*

(P/C15, comorbid neurodevelopmental and eating disorder)

There were several instances where a young person’s GP took on responsibility for their care when the young person did not meet the illness threshold for care elsewhere. For example, as described by the parent below, their GP spent a lot of time seeing the young person after his referral to a wellbeing service was rejected, which resulted in a positive experience of GP care:

*‘Dr* [name’s] *been brilliant and seen him every month. And she spends a load of time with him, which is why she’s always late with appointments, spends loads of time, usually books the last appointment for the day, spends sometimes 40 minutes with you, doesn’t she?’*

(P/C11, anxiety)

Having a long-term relationship with the same GP also led to positive experiences of accessing mental health support from the GP:

*‘She’s really supportive, but she knows the family, and been supportive of* [name’s] *mum and grandmother, and so she knows the circumstances surrounding the family.’*

(P/C11, anxiety)

## DISCUSSION

### Summary

This study is to the authors’ knowledge the first to explore the experiences of young people and their parents who accessed mental health support from primary care after falling through the gap post-CAMHS. Participants described variable levels of satisfaction, with some young people stating extremely positive experiences of accessing mental health support from their GP, whereas others, for various reasons, perceived their GPs as not supportive. Young people and their parents also identified struggling to navigate a disjointed care pathway. Facilitators for accessing mental health care in primary care included parents encouraging GP contact, having a long-term relationship with their GP, and the GP making time for the young person to speak to them. Conversely, identified barriers were seeing a new GP owing to moving away from home for university, anxiety caused by being in a GP waiting room, and thinking GPs do not understand mental illness.

Several participants found their GP was the only place they could go to access support for their mental health, as they were not eligible for care in AMHS or other community wellbeing services (for example, Improving Access to Psychological Therapies (IAPT). In some cases, GPs had made referrals to these services on behalf of the young person, only for them to be rejected. This leaves the GP responsible for that young person’s care despite them not being involved in the young person’s care since they made the initial referral to CAMHS, which in some cases will be over a decade before. The handover of care from CAMHS to the GP was also seen as problematic by several young people and their parents, where young people were ‘discharged to the GP’ without the GP having access to their notes or receiving any information about their further care from CAMHS. This exacerbated the disjointed nature of care pathways for some young people, where CAMHS signposted them to their GP to ask for a referral elsewhere, as opposed to CAMHS making a direct referral.

### Strengths and limitations

A particular strength of this study are the rich data gathered using a narrative interview approach, as most participants gave detailed accounts of their experiences. As this study interviewed young people and parents with different diagnoses across different NHS trusts, it was possible to gather a range of views across diverse settings. However, the sample is not representative in terms of ethnicity of participants, as almost all participants were white British. A further limitation was an absence of patient and public involvement in this research, owing to a lack of available funds. Some young people and parents were interviewed separately, and others preferred to be interviewed together. This was facilitated to ensure the young person felt comfortable during the interview process; however, it is noted that this may have influenced the responses of some young people in comparison with those who were interviewed alone.

### Comparison with existing literature

Young people in this study reported some positive experiences of visiting their GP for mental health support after CAMHS. A facilitator for accessing support from their GP found in this study was having a long-term relationship with the same doctor, something which was also identified in qualitative studies of young people’s experiences of primary care services.^25^^,^^26^ Young people have also highlighted that there is less stigma attached to receiving mental health support in primary care as opposed to attending a specialist AMHS.^27^

The finding that young people experienced problems with their medication after leaving CAMHS is consistent with the work of Newlove-Delgado *et al* ^5^^,^^11^ which found that young people with ADHD are at risk of the cessation of medication after leaving CAMHS, and that GPs described balancing the risks of prescribing ADHD medication without specialist input against the risk of the young person not receiving their medication. This study also found that GPs reported being involved in the young person’s care ‘by default’ after CAMHS ended, without sufficient handover of care.^11^ Challenges in the communication between CAMHS and the GP have also been reported in a qualitative study of GPs.^28^

This study identified problems faced by young people and parents who were ‘discharged to their GP’ by CAMHS without monitoring or support for mental health needs. This finding also supports the results of a systematic review of issues around GP care for young people with ADHD,^9^ indicating young people with other diagnoses experience similar problems after leaving CAMHS. Some young people and parents in this study felt that their GP did not fully understand mental illness, which mirrors the findings of a qualitative study which identified GP training needs around supporting young people with emotional distress.^29^

The findings from this study can be mapped onto the healthcare candidacy framework,^30^ which states that service users’ eligibility to access medical support is negotiated between the service user and the healthcare service. In this study, young people and parents attempted to navigate a complex care pathway to access support for their mental health after leaving CAMHS. In the context of seeking help when ‘falling through the gap’, young people asserted their candidacy by appearing at their GP to explain their need for support for their mental health. As identified, however, there were several barriers and facilitators which influenced this process.

### Implications for research and practice

These findings indicate the need for a managed transition of care for young people if their care is transferred to their GP after leaving CAMHS. This could involve a full handover of the young person’s notes by CAMHS, including any required actions for the GP and, potentially a pre-arranged GP appointment for the young person with a member of the CAMHS team. GPs can provide good continuity of care, as they often make the initial CAMHS referral and can have long-term relationships with young people and their families. The additional role schemes of mental health practitioners in primary care through the NHS Long Term Plan^31^ could also facilitate primary care based mental health support for young people after CAMHS care.

There needs to be improved communication between GPs and specialist care, both to facilitate onward referrals if required, and to provide support to GPs if the young person requires specialist medication. GPs should be aware of the challenges and needs of this population group and attempt to be proactive and make time to assess young people’s mental health needs if they take responsibility of care. This could be facilitated through further training for GPs to increase their knowledge of the mental health needs of transition-aged young people.

Future research should explore how primary care can meet the needs of young people who require mental health support after leaving CAMHS but are not eligible for specialist services. It is also crucial that any future research gains GP views on interventions for young people who have fallen through the gap and how care can be maintained in specialist services. However, as this research indicates the need for more integrated working across different services within the health system, studies should also consider exploring the views of mental health commissioners and managers to support improving the transition process and breaking down organisational barriers to providing good continuity of care. Future studies should also aim to explore the mental health service needs of young people from a diverse range of backgrounds. Experiences of young people and parents may have been affected by their diagnosis and time in services, which is not something that this study sought to examine. This needs further exploration in future work; for example, by looking at transition experiences in those with specific mental illnesses such as depression, psychosis, or eating disorders.
